# Demographic Patterns and Clinical Presentations of Demodicosis in a Longitudinal Study From Khuzestan Province, Southwest Iran

**DOI:** 10.1002/hsr2.71529

**Published:** 2025-11-17

**Authors:** Sharif Maraghi, Mehdi Tavalla, Abdollah Rafiei, Mohammad Javad Boozhmehrani, Akbar Hoseinnejad

**Affiliations:** ^1^ Department of Parasitology and Mycology, School of Medicine Islamic Azad University Ahvaz Iran; ^2^ Iran Zamin Medical Diagnostic Laboratory Naderi Grand Medical Complex Naderi Street Ahwaz Iran; ^3^ Department of Parasitology, School of Medicine Ahvaz Jundishapur University of Medical Sciences Ahvaz Iran; ^4^ Infectious and Tropical Diseases Research Center, Health Research Institute Ahvaz Jundishapur University of Medical Sciences Ahvaz Iran; ^5^ Student Research Committee Ahvaz Jundishapur University of Medical Sciences Ahvaz Iran; ^6^ Department of Medical Mycology, School of Medicine Ahvaz Jundishapur University of Medical Sciences Ahvaz Iran

**Keywords:** co‐infections, *Demodex* mites, demodicosis, dermatology, epidemiology

## Abstract

**Background and Aims:**

Demodicosis is a significant dermatological condition caused by *Demodex* mites, with a wide range of clinical manifestations. Despite its prevalence, population‐based studies on the epidemiology, co‐infections, and atypical presentations of demodicosis remain limited globally. This study aimed to investigate the demographic patterns, clinical characteristics, and associated co‐infections of demodicosis over a 22‐year period in southwest Iran.

**Methods:**

A total of 382 patients with confirmed demodicosis were retrospectively analyzed. Data were collected from records of Iran Zamin Medical Diagnostic Laboratory (2002–2024). Demographic characteristics, clinical presentations, co‐infections, and atypical manifestations were documented. Skin scrapings were examined microscopically to confirm *Demodex* infestation, and statistical analyses were conducted to identify significant patterns.

**Results:**

The mean patient age was 36.6 ± 12.8 years, with a marked female predominance (77.7%). The age distribution showed that the 21–35 age group accounted for the largest proportion of cases (45.3%), while pediatric (≤ 20 years) and geriatric (> 65 years) groups showed no significant gender disparity. Co‐infections were identified in 6.8% of cases, predominantly involving dermatophyte infections, such as *Tinea facei* (46.1% of co‐infections). This study also highlights novel co‐infections and atypical presentations, including involvement of the trunk, sinus, ear, and thigh.

**Conclusion:**

This is the first large‐scale study of demodicosis in the general population of Iran, covering a 22‐year period. It is also among the first worldwide to explore co‐infections systematically. The findings emphasize significant demographic and clinical patterns, particularly age‐ and gender‐specific susceptibility and the complex interplay of co‐infections. These insights expand the understanding of demodicosis and highlight the importance of considering its atypical manifestations and associated infections in clinical practice.

## 1 Introduction

Demodicosis represents a significant dermatological condition caused by *Demodex* mites (Class Arachnida, Subclass Acarina), which are obligate human ectoparasites found across diverse ethnic populations worldwide [1]. Two species primarily affect humans: *Demodex folliculorum* and *D. brevis*, each with distinct morphological characteristics and habitat preferences within the skin structure [2]. While *D. folliculorum* predominantly inhabits hair follicle infundibula and reaches 279–294 µm in length, *D. brevis* is smaller (165–208 µm) and penetrates deeper into sebaceous glands and meibomian glands [3].

These mites exhibit a predilection for sebum‐rich areas, particularly the T‐zone of the face (forehead, cheeks, nose, and nasolabial folds), though they can colonize various body regions, including the scalp, external ear, upper chest, mons pubis, and buttocks [4]. Their life cycle spans 14–18 days, progressing through egg, larval, protonymphal, and deutonymphal stages before reaching maturity [5]. The parasites sustain themselves on sebum and epithelial cells from follicles and glands, potentially triggering inflammatory responses in the skin and eyelid margins [6].

Clinical manifestations of demodicosis are diverse, encompassing rosacea‐like conditions, follicular pitiriasis, pustular folliculitis, perioral granulomatous dermatitis, and facial hyperpigmentation [7]. In the eyelids, chronic blepharitis may develop due to follicular obstruction and inflammatory responses to mite metabolites and chitinous remnants [8]. Typically, infected follicles harbor 2–6 mites, though higher numbers can occur [9]. These parasites may also serve as vectors for various microorganisms, including *Streptococcus*, *Staphylococcus*, and notably *Bacillus oleronius*, whose antigens can stimulate host inflammatory responses and affect peripheral blood mononuclear cell proliferation [1, 2].

Transmission occurs through direct skin contact or exposure to dust containing eggs, with certain populations facing elevated risk. Healthcare workers and medical students, due to frequent patient contact, may experience higher exposure rates. Additionally, immunocompromised individuals, including those with HIV infection, show increased susceptibility to demodicosis. Despite its clinical significance, precise prevalence data remain limited, particularly regarding ocular involvement [10, 11].

Current research gaps include understanding the factors influencing gender‐specific susceptibility, age‐related distribution patterns, and the relationship between demodicosis and concurrent skin conditions. Our study addresses these knowledge gaps by analyzing a large‐scale, long‐term data set from Khuzestan province, Iran, focusing on demographic patterns, co‐infections, and clinical presentations across different patient populations.

## 2 Materials and Methods

### 2.1 Study Design and Ethical Considerations

This cross‐sectional observational study was conducted at Iran Zamin Medical Diagnostic Laboratory, Khuzestan province, Iran. The study protocol was approved by the Ethics Committee of Ahvaz Jundishapur University of Medical Sciences (IR.AJUMS.REC.1403.188) and conducted in accordance with the Declaration of Helsinki. Written informed consent was obtained from all participants or their legal guardians for minors.

### 2.2 Study Population and Setting

We retrospectively analyzed the medical records of patients with confirmed *Demodex* infestation at Iran Zamin Medical Diagnostic Laboratory over a 22‐year period (2002–2024). These cases were selected from a larger pool of individuals presenting with dermatological manifestations suggestive of dermatophytosis, rosacea, or undiagnosed dermatitis. The laboratory serves as a major diagnostic center for various cities within Khuzestan province, providing a representative sample of the region's population.

### 2.3 Clinical Assessment and Data Collection

Demographic data, including age, gender, and clinical presentations, were recorded using standardized forms. Detailed medical histories were obtained, including previous treatments and concurrent conditions. Physical examinations were performed by trained dermatologists, focusing on the characteristics and distribution of skin lesions.

### 2.4 Sampling and Laboratory Procedures

Skin scrapings were collected from affected areas using sterile surgical blades. For facial lesions, samples were obtained from areas showing erythema, papules, or pustules. For atypical presentations (trunk, thigh, groin, ear discharge, and sinus), specimens were collected from the affected sites following standard dermatological protocols.

### 2.5 Sample Processing

**Figure 1:**
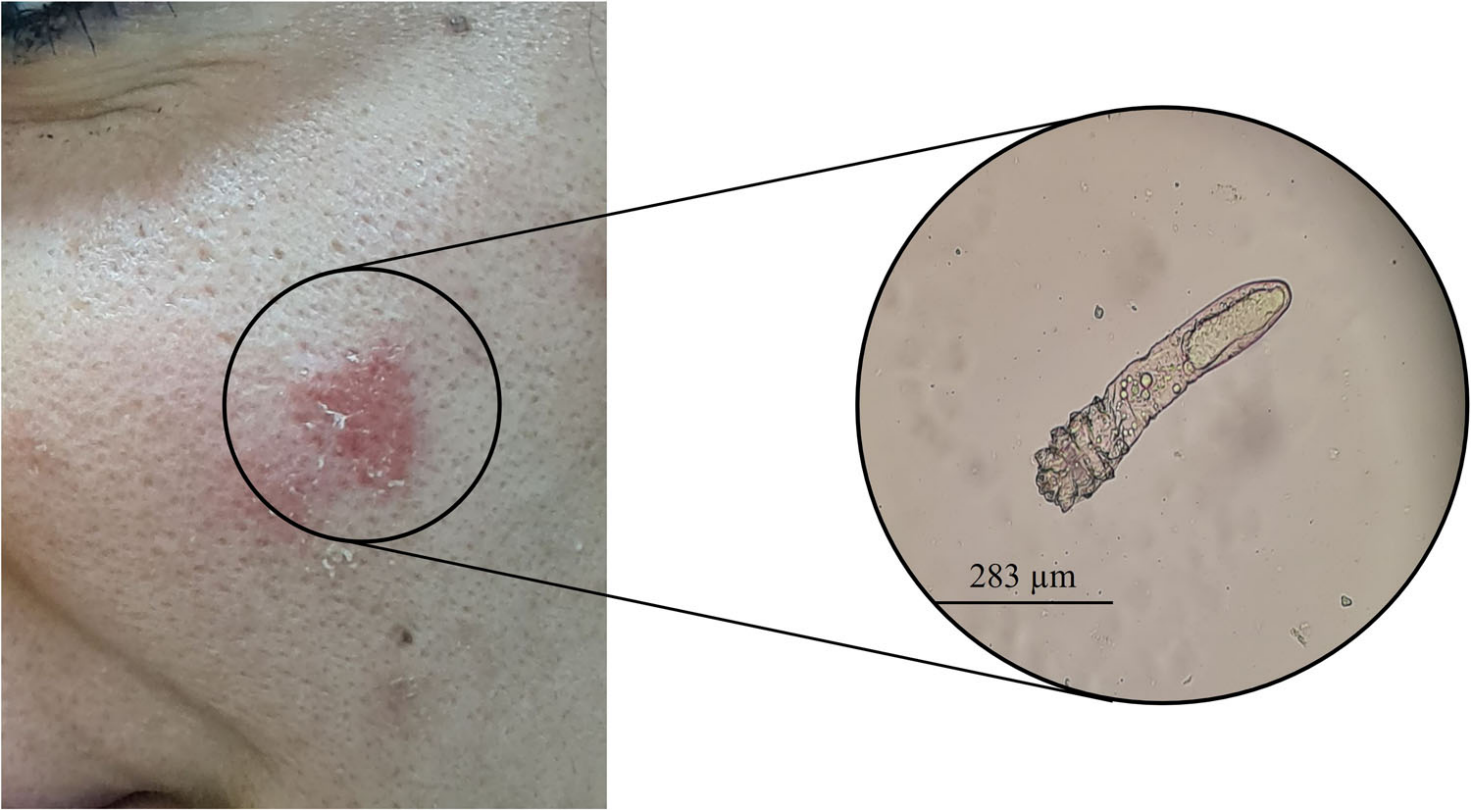
Clinical and microscopic presentation of demodicosis. Left: Characteristic erythematous lesion on facial skin showing inflammatory response consistent with demodicosis. Right: Light microscopy image (10× magnification) of a skin scraping from the affected area showing a *Demodex* specimen approximately 283 μm in length. The mite displays morphological features suggestive of *D. folliculorum*, including an elongated opisthosoma with annulated (ring‐like) appearance and a semi‐transparent prosoma.

Specimens were processed using a standardized protocol. Skin scrapings were placed on clean glass slides and treated with 20% potassium hydroxide (KOH) solution to dissolve keratin and facilitate visualization of potential pathogens. Preparations were examined under light microscopy at ×10 and ×40 magnifications (Figure 1).

*Demodex* species were identified based on their characteristic morphology, including body size, segmentation, and localization within the follicular material. In addition, co‐infections were systematically assessed during microscopic evaluation. Dermatophyte infections were identified by the presence of hyaline, septate hyphae and, when present, arthroconidia. Yeast‐like fungi (e.g., *Candida* spp.) were recognized by budding yeast cells or pseudohyphae. Non‐dermatophyte fungal elements were recorded when morphologically distinguishable.

### 2.6 Quality Control

All microscopic examinations were conducted by experienced laboratory technicians following standardized protocols. For validation purposes, 10% of samples were randomly selected and re‐examined by a second observer, and any discrepancies in findings were resolved through consensus discussion among the laboratory staff. The morphological identification of *Demodex* mites was based on established diagnostic criteria and verified through photographic documentation when available.

### 2.7 Statistical Analysis

Statistical analyses were performed using R version 4.2.0 (R Foundation for Statistical Computing, Vienna, Austria). Descriptive statistics were calculated for demographic characteristics, including means, standard deviations, and frequency distributions. *χ* ^2^ tests were used to analyze gender distribution against an expected 1:1 ratio for the overall population, age groups, co‐infections, and atypical presentations where sample size permitted (*n* ≥ 5). Age groups were defined as ≤ 20 (pediatric and adolescent), 21–35 (young adult), 36–50 (middle adult), 51–65 (late adult), and > 65 years (elderly) based on clinically relevant cutoffs. For age‐related analyses, we calculated mean ± standard deviation, while categorical variables were expressed as frequencies and percentages. Fisher's exact test was applied for subgroup analyses with expected frequencies less than 5. All statistical tests were two‐sided, and a *p* value < 0.05 was considered statistically significant. For proportion calculations of specific conditions and presentations, 95% confidence intervals were determined using the Wilson score method. Statistical testing was not performed on subgroups with fewer than five cases to avoid potential type II errors.

## 3 Results

Our analysis of 382 demodicosis cases collected over a 22‐year period from Khuzestan province revealed significant patterns in both age and gender distribution (Table 1). The age of patients ranged from 8 months to 81 years, with a mean age of 36.6 ± 12.8 years. Overall, females constituted 77.7% (297/382) of cases compared to 22.3% (85/382) males, representing a highly significant gender disparity (*χ*² = 117.65, *p* < 0.001). Microscopic examination of skin scrapings revealed *Demodex* mites with morphological characteristics and size measurements most consistent with *D. folliculorum* (Figure 1).

**Table 1:** Demographic and clinical characteristics of demodicosis cases in Khuzestan province.

Characteristic	Total (*n*, %)	Gender		Statistical analysis
		Female (*n*, %)	Male (*n*, %)	
Overall	382 (100)	297 (77.7)	85 (22.3)	*χ*² = 117.65, *p* < 0.001
Age groups (years)				
≤ 20	36 (9.4)	21 (58.3)	15 (41.7)	*χ*² = 1.00, *p* = 0.32
21‐35	173 (45.3)	139 (80.3)	34 (19.7)	*χ*² = 63.73, *p* < 0.001
36‐50	118 (30.8)	96 (81.4)	22 (18.6)	*χ*² = 46.41, *p* < 0.001
51‐65	44 (11.5)	36 (81.8)	8 (18.2)	*χ*² = 17.82, *p* < 0.001
> 65	11 (2.8)	5 (45.5)	6 (54.5)	*χ*² = 0.09, *p* = 0.76
Co‐infections	26 (6.8)	19 (73.1)	7 (26.9)	*χ^2^* = 5.54, *p* = 0.019
*T. facei*	12 (46.1)	10 (83.3)	2 (16.7)	*χ*² = 5.33, *p* = 0.021
*T. corporis*	8 (30.7)	6 (75.0)	2 (25.0)	*χ*² = 2.00, *p* = 0.16
*T. versicolor*	3 (11.5)	2 (66.7)	1 (33.3)	*
*T. cruris*	1 (3.9)	1 (100.0)	0 (0)	*
Cutaneous leishmaniasis	1 (3.9)	0 (0)	1 (100.0)	*
Intertriginous candidiasis	1 (3.9)	0 (0)	1 (100.0)	*
Atypical presentations	7 (1.8)	4 (57.1)	3 (42.9)	*χ*² = 0.14, *p* = 0.71
Sinus involvement	2 (28.6)	0 (50.0)	2 (50.0)	*
Trunk involvement	2 (28.6)	1 (50.0)	1 (50.0)	*
Other sites**	3 (42.8)	2 (66.7)	1 (33.3)	*
Systemic conditions	2 (0.5)	2 (100.0)	0 (0)	*
Lupus erythematosus	1 (50.0)	1 (100.0)	0 (0)	*
Acute lymphoid leukemia	1 (50.0)	1 (100.0)	0 (0)	*

*Note:* Percentages may not total 100 due to rounding. *χ*2 tests were performed for gender distribution against the expected 1:1 ratio overall and within each age group.

* Sample size too small for reliable *χ* ^2^ analysis (*n* < 5).

** Other sites include ear, thigh, and groin involvement (*n* = 1 each).

Age distribution analysis demonstrated a nonrandom pattern, with the highest distribution observed in the 21–35 age group (45.3% of cases). The age‐specific gender analysis revealed that the female predominance was not uniform across age groups (Table 1). While younger (≤ 20 years) and older (> 65 years) age groups showed no significant gender disparity (58.3% vs. 41.7%, *χ*² = 1.00, *p* = 0.32; and 45.5% vs. 54.5%, *χ*² = 0.09, *p* = 0.76, respectively), the middle age groups demonstrated marked female predominance. This disparity was most pronounced in the 21–35 age group (80.3% female, *χ*² = 63.73, *p* < 0.001), followed by the 36–50 age group (81.4% female, *χ*² = 46.41, *p* < 0.001), and the 51–65 age group (81.8% female, *χ*² = 17.82, *p* < 0.001).

**Figure 2:**
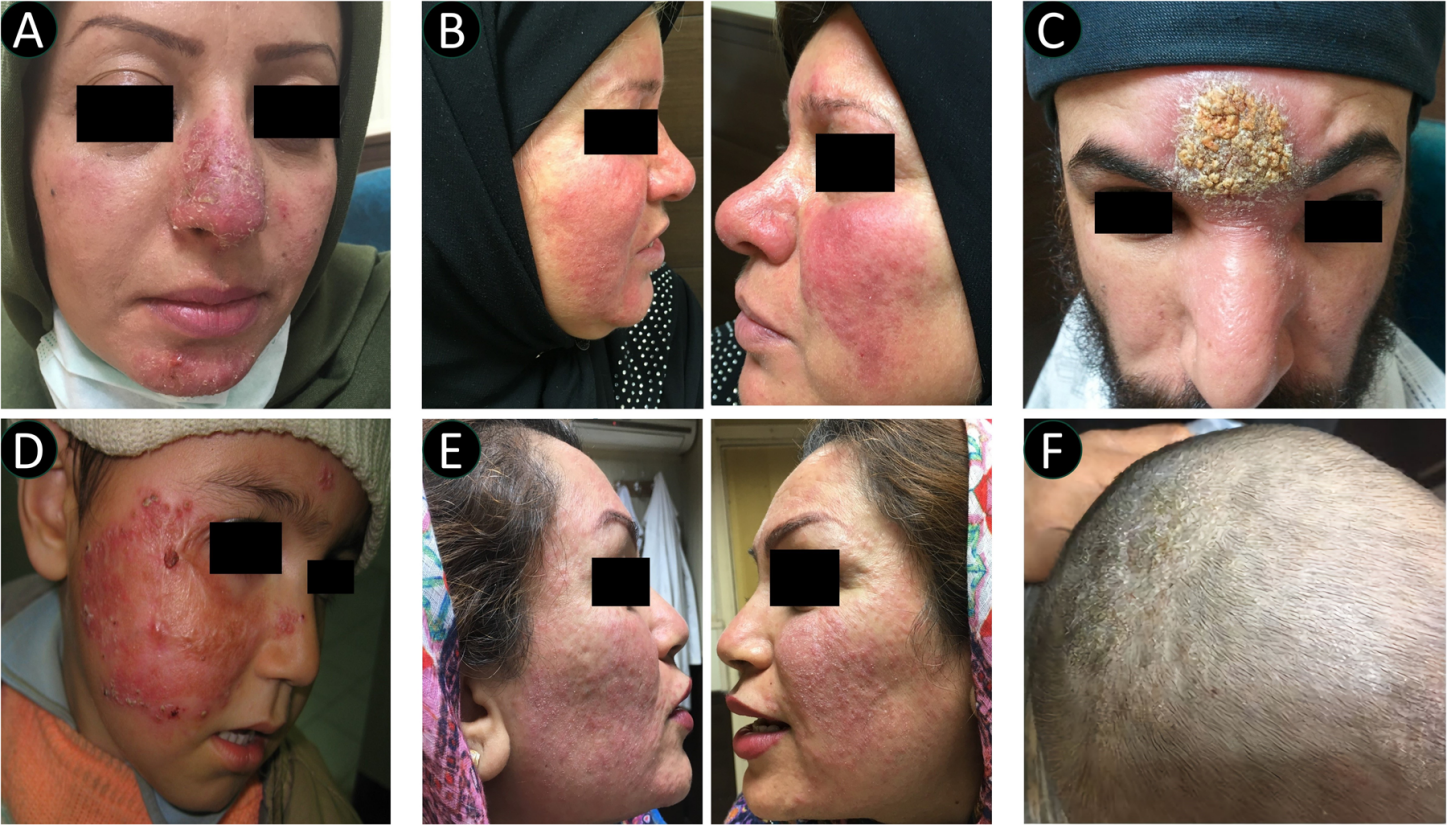
Clinical presentations of demodicosis and its variants. (A) Crusted demodicosis in a young female patient showing characteristic facial lesions. (B) Bilateral facial profiles demonstrating rosacea‐like demodicosis with typical inflammatory features. (C) Co‐infection of demodicosis and cutaneous leishmaniasis in a male patient, illustrating complex disease interaction. (D) Demodicosis manifestation in a pediatric patient with acute lymphoblastic leukemia, demonstrating association with immunocompromised status. (E) Bilateral facial profiles showing diffuse pattern of demodicosis in female patients, representing typical distribution pattern. (F) Unusual presentation of scalp demodicosis in a male pediatric patient, illustrating atypical location of infestation.

Co‐infections were documented in 26 cases (6.8%), predominantly involving various forms of *tinea*, with a significant female predominance (73.1% female, *χ*² = 5.54, *p*= 0.019) (Table 1). *T. facei* was the most common co‐infection (12 cases, 46.1% of co‐infections), showing significant female predominance (83.3% female, *χ*² = 5.33, *p* = 0.021), followed by *T. corporis* (8 cases, 30.7%) with a nonsignificant gender distribution (75.0% female, *χ*² = 2.00, *p* = 0.16), *T. versicolor* (3 cases, 11.5%), and *T. cruris* (1 case, 3.9%). Other co‐infections included cutaneous leishmaniasis and intertriginous candidiasis (1 case each, both male) (Figure 2).

Notably, we observed seven cases (1.8%) of atypical presentations, including sinus involvement (2 cases), trunk involvement (2 cases), and single cases each of ear, thigh, and groin involvement. These atypical presentations showed a more balanced gender distribution (57.1% female vs. 42.9% male, *χ*² = 0.14, *p*= 0.71), significantly different from the typical presentation pattern. Systemic conditions were documented in two cases (0.5%), both female patients, including one case each of lupus erythematosus and acute lymphoid leukemia (Figure 2).

## 4 Discussion

Our 22‐year study reveals distinct demographic patterns in demodicosis presentation, particularly regarding gender distribution and age‐related susceptibility. We observed a marked female predominance (77.7%) during reproductive years, with the most pronounced gender disparity occurring in three distinct age groups: 21–35 years (80.3%), 36–50 years (81.4%), and 51–65 years (81.8%). Notably, this gender difference becomes statistically insignificant in both pediatric (≤ 20 years, 58.3%) and geriatric populations (> 65 years, 45.5%), suggesting age‐specific pathophysiological mechanisms.

While the retrospective nature of our study does not allow definitive determination of the cause of gender differences, we hypothesize that the higher proportion of female cases may partly reflect healthcare‐seeking behavior, as women are statistically more likely to seek medical attention for dermatological concerns. These findings partially align with recent European studies, particularly research from Greece demonstrating similar female predominance (70% female cases, 30% male cases) in demodicosis [12]. However, our results contrast with findings from northern Poland, where male predominance was reported [13].

Several biological factors may contribute to the observed gender disparity. Given that *Demodex* species are part of the normal skin microbiota, their presence alone does not necessarily indicate pathology. However, disruption of the host‐parasite balance, particularly in conditions of immune dysregulation or altered skin microbiome, can lead to clinical manifestations [14, 15]. Hormonal influences likely play a crucial role in this balance, particularly given the correlation between reproductive years and the higher proportion of female cases. *D. folliculorum*, the predominant species in our study, thrives in sebum‐rich environments [16]. The well‐documented influence of female reproductive hormones on sebum production may create more favorable conditions for mite colonization during reproductive years [17, 18]. Additionally, studies have demonstrated that hormones such as prolactin and progesterone can modulate immune responses, potentially affecting host–parasite interactions through Th1/Th2 balance shifts and immunoregulatory mechanisms [19, 20, 21].

Behavioral and sociocultural factors may also contribute to these findings. Women's greater likelihood to seek dermatological care and more frequent use of cosmetic products could influence both detection rates and mite colonization patterns. The increased use of skin treatments and exfoliating products among women may alter the skin microenvironment, potentially affecting *Demodex* populations [22].

The absence of gender disparity in pre‐pubertal and post‐menopausal populations provides compelling evidence for the role of reproductive hormones in disease manifestation. These findings suggest that the pathophysiology of demodicosis varies significantly across different life stages and between genders, potentially influenced by a complex interplay of hormonal, immunological, and behavioral factors.

Our data revealed distinct age distribution patterns across the study population. The pediatric age group (≤ 20 years) represented only 9.4% of all cases, while young adults (21–35 years) constituted the largest group at 45.3% of cases. Middle‐aged adults (36–50 years) accounted for 30.8% of cases, followed by a gradual decline in the distribution of cases, with 11.5% in the 51–65 age group and 2.8% in those over 65 years. These age‐related patterns indicate varying susceptibility to demodicosis across different life stages, with the mean age of affected individuals being 36.6 ± 12.8 years. Several hypotheses may explain the predominance of young adults in our cohort. Hormonal influences during reproductive years, particularly the impact of sex hormones on sebum production, may create a favorable microenvironment for mite proliferation. In addition, the widespread use of cosmetic products in this age group could alter the skin barrier and microbiome, thereby facilitating infestation. Immune status may also play a role, as subtle immunomodulatory changes in young adults could influence host–parasite interactions. Finally, occupational exposures and lifestyle habits common in this demographic might contribute to a higher risk. These factors, taken together, may account for the peak proportion observed in our study population. The large‐scale study from northern Poland corroborates our findings regarding low prevalence in children; however, they reported higher prevalence rates in elderly populations. This discrepancy might be attributed to healthcare‐seeking behaviors in our study region, where elderly individuals may be less likely to seek medical attention for dermatological symptoms compared to younger patients. Numerous studies have consistently demonstrated the significant impact of age on demodicosis prevalence, supporting our age‐related patterns [5, 23, 24].

Our study documented co‐infections in 6.8% of cases, with dermatophyte infections predominating. The majority of these co‐infections presented as *T. facei* (46.1% of co‐infections), followed by *T. corporis* (30.7%), *T. versicolor* (11.5%), and *T. cruris* (3.9%). The predominance of dermatophytes, particularly *T. facei*, may be explained by altered local immunity secondary to *Demodex*‐associated inflammation that facilitates fungal colonization, or by shared predisposing factors such as humid environments and hygiene practices. Additionally, we observed cases of intertriginous candidiasis and cutaneous leishmaniasis. This pattern of co‐infections is particularly noteworthy as it complicates both diagnosis and clinical management. While nonspecific skin manifestations of demodicosis often lead to diagnostic challenges, the presence of concurrent infections can further obscure the clinical picture and potentially exacerbate symptoms.

The relationship between *Demodex* and co‐infections merits particular attention. Although *Demodex* mites are commonly considered commensal organisms, their interaction with concurrent infections may alter the inflammatory response and disease severity [15]. The significant frequency of facial dermatophyte infections coinciding with demodicosis suggests potential synergistic pathogenic mechanisms or shared predisposing factors. To our knowledge, this represents the first large‐scale epidemiological study systematically documenting co‐infections in demodicosis, as previous reports have been limited to individual case studies. This gap in the literature highlights the need for further investigation into the clinical significance and management implications of these co‐infections. Recent evidence supports the view that *Demodex* mites can act as pathogenic agents under specific conditions rather than remaining harmless commensals. Host immune responses, such as cytokine release, activation of TLR2 pathways, and the involvement of bacterial vectors, are thought to play an important role in ocular and cutaneous disease [25]. The interaction between mite overgrowth and microbial factors may increase disease severity and help to explain the clinical variability observed in our study population.

A significant finding of our study was the documentation of atypical presentations (1.8% of cases), which included diverse anatomical locations beyond the traditional facial distribution of *Demodex*. These atypical manifestations included sinus involvement (28.6% of atypical cases), trunk involvement (28.6%), and other sites, including ear, thigh, and groin (42.8%). The identification of these non‐facial presentations challenges the conventional understanding of demodicosis distribution and emphasizes the importance of considering this diagnosis in unexpected locations. Notably, these atypical presentations showed a more balanced gender distribution compared to typical facial involvement, suggesting potentially different pathophysiological mechanisms. Such atypical localizations are noteworthy as they may arise from dissemination, local skin environment changes, or prior interventions, and can easily be misdiagnosed as other dermatoses.

The recognition of these varied presentations has important diagnostic implications. Given that most cases in our study population had received previous misdiagnoses and inappropriate treatments, our findings underscore the necessity for increased clinical awareness of both typical and atypical manifestations of demodicosis. This is particularly relevant when evaluating patients with resistant or recurrent dermatological conditions that have failed conventional treatments. These observations expand our understanding of the clinical spectrum of demodicosis and suggest the need for a more comprehensive approach to patient examination and diagnostic consideration.

In summary, our study highlights three major findings that warrant particular emphasis. First, we observed a distinctive demographic profile characterized by a young adult predominance and a significant female skew, particularly during reproductive years. Second, although co‐infections were relatively uncommon, they were consistently present, with dermatophyte infections emerging as the predominant concurrent condition. Third, we documented atypical anatomical sites of involvement, including the trunk, sinus, ear, and thigh, which challenge the conventional understanding of demodicosis distribution.

While our study provides valuable long‐term data, several limitations should be considered. The retrospective nature of the study and potential referral bias may affect the generalizability of findings. The lack of hormonal measurements and detailed immune status assessment limits our ability to definitively establish causal relationships. Additionally, the single‐center design may not fully represent the broader population patterns. Although preliminary microscopic observations suggested *D. folliculorum* as the predominant species, detailed species‐level analysis was not pursued as the primary diagnostic goal was rapid identification to facilitate timely therapeutic intervention. This precluded precise determination of species distribution patterns and potential species‐specific clinical correlations. Furthermore, some of the observed co‐infection patterns, while not previously reported, should be interpreted with caution as they may represent incidental rather than causal associations.

## 5 Conclusion

This 22‐year study provides one of the most comprehensive overviews of demodicosis in the Iranian population. The findings highlight that the disease is not restricted to facial involvement and may also arise in less expected anatomical sites, which requires clinicians to maintain a high index of suspicion beyond classical facial lesions. The documentation of co‐infections further suggests that *Demodex* may interact with other pathogens in ways that complicate diagnosis and management. Altogether, our results call for greater clinical awareness, systematic screening approaches, and future research to clarify the host, immune, and environmental factors that shape the epidemiology of demodicosis.

## Author Contributions

**Sharif Maraghi:** methodology, conceptualization, supervision, writing – review and editing, and data curation. **Mehdi Tavalla:** methodology, conceptualization, writing – review and editing, supervision, and project administration. **Abdollah Rafiei:** methodology, supervision, writing – review and editing. **Mohammad Javad Boozhmehrani:** writing – original draft, writing – review and editing, software, visualization, and formal analysis. **Akbar Hoseinnejad:** writing – original draft and formal analysis.

## Consent

We confirm that written informed consent was obtained from all patients whose images are included in this article. The consent process involved explaining the purpose of using their images in this study, the extent of their publication, and assurances that their identities and personal information would remain confidential.

## Conflicts of Interest

The authors declare no conflicts of interest.

## Transparency Statement

The corresponding authors, Mehdi Tavalla and Mohammad Javad Boozhmehrani, affirm that this manuscript is an honest, accurate, and transparent account of the study being reported; that no important aspects of the study have been omitted; and that any discrepancies from the study as planned (and, if relevant, registered) have been explained.

## Data Availability

The data sets used and/or analyzed during the current study are available from the corresponding author on reasonable request.
